# Genotyping of Clinical *Mycobacterium tuberculosis* Isolates Based on IS*6110* and MIRU-VNTR Polymorphisms

**DOI:** 10.1155/2013/865197

**Published:** 2013-12-17

**Authors:** Anna Żaczek, Anna Brzostek, Arkadiusz Wojtasik, Jarosław Dziadek, Anna Sajduda

**Affiliations:** ^1^Department of Biochemistry and Cell Biology, Faculty of Biology and Agriculture, University of Rzeszów, Ćwiklińskiej 2, 35-601 Rzeszów, Poland; ^2^Institute of Medical Biology, Polish Academy of Sciences, Lodowa 106, 93-232 Łódź, Poland; ^3^Proteon Pharmaceuticals, Tylna 3a, 90-364 Łódź, Poland; ^4^Department of Microbial Genetics, Faculty of Biology and Environmental Protection, University of Łódź, Banacha 12/16, 90-237 Łódź, Poland

## Abstract

In this study, 155 clinical *Mycobacterium tuberculosis* isolates were subject to genotyping with fast ligation-mediated PCR (FLiP). This typing method is a modified mixed-linker PCR, a rapid approach based on the PCR amplification of *Hha*I restriction fragments of genomic DNA containing the 3′ end of IS*6110* and resolving the amplicons by polyacrylamide gel electrophoresis. The results were compared with previous data of the more commonly used methods, 15-locus mycobacterial interspersed repetitive unit-variable number tandem repeat (MIRU-VNTR) typing and, to verify combined FLiP/MIRU-VNTR clusters, the reference IS*6110* restriction fragment length polymorphism (RFLP). FLiP banding patterns were highly reproducible and polymorphic. This method differentiated 119 types among the study set compared to 108 distinct MIRU-VNTR profiles. The discriminatory power of FLiP was slightly higher than that of MIRU-VNTR analysis (Hunter-Gaston Discriminatory Index = 0.991 and 0.990, resp.). Detailed comparison of the clusters defined by each of the methods revealed, however, a more apparent difference in the discriminatory abilities that favored FLiP. Clustering of strains by using combined results of these two PCR-based methods correlated well with IS*6110* RFLP-defined clusters, further confirming high discriminatory potential of FLiP typing. These results indicate that FLiP could be an attractive and valuable secondary typing technique for verification of MIRU-VNTR clusters of *M. tuberculosis* strains.

## 1. Introduction

Tuberculosis (TB) is an infectious disease caused by *Mycobacterium tuberculosis*, one of the most dangerous human pathogens. In 2009 almost two million people worldwide died of TB [1]. Recently, molecular typing of *M. tuberculosis* has greatly improved our knowledge of TB epidemiology and allowed for a better control of this disease [2]. Identification of epidemiologically linked *M. tuberculosis* strains helps to reveal the source of infection, to trace the transmission routes of various strains, and to determine the risk factors for TB transmission in a community. Molecular epidemiology enables distinguishing between exogenous reinfection and endogenous reactivation, thus helping in a more effective elimination of TB from the population. In a laboratory, molecular methods can be used to identify cross-contamination [3–5].

Various genetic markers are currently used in molecular epidemiology of TB. A high degree of DNA polymorphism in *M. tuberculosis* strains is associated with repetitive DNA elements such as insertion sequences (IS) and short repetitive DNA sequences [2]. The insertion sequence IS*6110 *is especially useful in genotyping of *M. tuberculosis* [6]. IS*6110 *restriction fragment length polymorphism (RFLP) is one of the methods based on the variability of this element and is the current international typing standard in the epidemiology of TB [7]. The reference method relies on the analysis of the number of IS*6110* copies and their locations within genomes of *M. tuberculosis* strains and shows the highest discriminatory potential on the population level [2]. However, IS*6110* RFLP is labor-intensive and expensive and requires high quantities of purified genomic DNA (at least 2 *μ*g). Moreover, it is not applicable to the analysis of *M. tuberculosis* strains with low copy numbers of IS*6110* or strains devoid of this element [8].

Due to those disadvantages of the IS*6110* RFLP, alternative PCR-based methods have been developed. They are easy to perform, require small amounts of genomic DNA, and can be performed even on nonviable organisms or directly from clinical specimens, thus reducing the time, cost and labor-intensity required for the analysis [8–10]. One of these genotyping methods exploits polymorphism in the variable number of tandem repeats (VNTRs) of mycobacterial interspersed repetitive units (MIRUs) in the genomes of *M. tuberculosis* strains [11]. Out of the 41 identified loci that contain MIRU repeats, 12, 15, or 24 the most variable sequences have been frequently used for differentiation of *M. tuberculosis* strains, based on the number of repeats in each locus investigated [12–14]. MIRU-VNTR typing has discriminatory potential close to that of the reference method or even higher in the case of isolates with low IS*6110* copy numbers [15].

Fast ligation-mediated PCR (FLiP) is another method based on IS*6110* polymorphism [16]. FLiP is a modified mixed-linker PCR, a rapid typing method based on the PCR amplification of RFLP fragments containing the 3′ end of IS*6110* and resolving the amplicons by polyacrylamide gel electrophoresis [17]. Compared with eight other typing methods for *M. tuberculosis* complex, the FLiP method shows similar high discriminatory power and reproducibility [18]. However, despite its ability to reliably differentiate between strains, FLiP has not been frequently used since its publication.

In this context, we extend our earlier, preliminary observations [19, 20] and present here the results on genotyping of 155 clinical *M. tuberculosis* isolates using FLiP in comparison with previously performed 15-locus MIRU-VNTR typing [21]. Clusters identified in both PCR-based methods were further analyzed by the reference IS*6110* RFLP.

## 2. Materials and Methods

### 2.1. Bacterial Isolates

The 155 *M. tuberculosis* isolates, of 234 isolates previously studied [21], were available for the present analysis. They were obtained in 2005–2008 from 153 TB patients diagnosed at the Center for Lung Diseases Treatment and Rehabilitation in Łódź, Poland. In two patients, a second isolate was obtained in a time interval and those two repetitive isolates were also included in the present study. The strains were cultured on Löwenstein-Jensen slants from sputum (*n* = 126), bronchial aspirate (*n* = 15), throat swab (*n* = 7), pleural fluid (*n* = 4), larynx swab (*n* = 1), gum pus (*n* = 1), and urine (*n* = 1). All the isolates were tested for their susceptibility to isoniazid (INH), streptomycin (SM), ethambutol (EMB), and rifampin (RMP) using the BACTEC 460-TB system (Becton-Dickinson, Sparks, MD, USA). Only six (3.9%) strains were resistant to at least one anti-TB drug: INH (*n* = 3); SM (*n* = 1); INH and RMP (*n* = 1); INH, RMP and SM (*n* = 1).

Genomic DNA was extracted and purified from all the isolates using the protocol by van Embden et al. [7], recommended for the standard IS*6110* RFLP typing. The concentration of DNA was measured with the NanoDrop ND-1000 spectrophotometer (NanoDrop Technologies, Montchanin, DE, USA) prior it was used in molecular typing.

### 2.2. Fast Ligation-Mediated PCR

FLiP was performed as originally described by Reisig et al. [16]. Briefly, the linker was synthesized by annealing of the oligonucleotides NLO (5′-GCATTTGAATTCCACGTCAGCGACTGCACG-3′) and BA (5′-TGCAGUCGCUGACGU GGAA-3′). The oligonucleotides were added in equimolar amounts into 100 *μ*L 1 × Gold Buffer (Invitrogen) and heated to 94°C for 15 min, followed by three cycles with 58°C for 10 min and 70°C for 5 min. Next, the DNA samples were digested with 0.5 U/*μ*l *Hha*I (Fermentas Life Technologies) and the restriction fragments were ligated to the linker (0.67 mM) using 0.05 U/*μ*L T4 DNA ligase in 20 *μ*L 1 × ligation buffer (Fermentas Life Technologies). Both reactions were performed simultaneously for 2 h at 25°C. Five *μ*L of the restriction-ligation mixture was used in amplification reaction. The PCR mix (50 *μ*L) contained primers IS54 (5′-TCGACTGGTTCAACCATCGCCG-3′) and Flip1 (5′-TTTGAATTCCACGTCAGCGACTG C-3′) (12.5 pM each), 1.25 mM MgCl_2_, 0.8 mM deoxynucleoside triphosphates, 0.5 U uracil DNA glycosylase (UDG, Invitrogen), and 2.5 U Ampli*Taq* Gold DNA polymerase (Invitrogen). The reaction began with an incubation at 50°C for 10 min to cut BA oligonucleotide of the linker with UDG. The cycling conditions consisted of an activation of Ampli*Taq* Gold DNA polymerase at 95°C for 10 min, followed by 30 cycles with 30 s at 69°C and 1 min at 72°C, and a final extension for 7 min at 72°C. Each experiment included negative (sterile ultrapure water) and positive (DNA of *M. tuberculosis* H37Rv) controls processed together with the test samples.

PCR products were resolved by 8% polyacrylamide gel electrophoresis. Banding patterns were visualized by ethidium bromide staining and photodocumented under UV light.

### 2.3. Genotype Analysis

The FLiP patterns were examined visually and then subject to computer-assisted analysis with BioNumerics 5.0 (Applied Maths, Sint-Martens-Latem, Belgium). The similarity (percentage) of patterns was calculated by the unweighted pair group method with arithmetic averages (UPGMA) and Dice similarity coefficient. A cluster was defined as a group of at least two isolates showing 100% identical DNA fingerprints.

The Hunter-Gaston Discriminatory Index (HGDI) [22] was used as a numerical index for the discriminatory power of the typing methods.

## 3. Results

**Figure 1:**
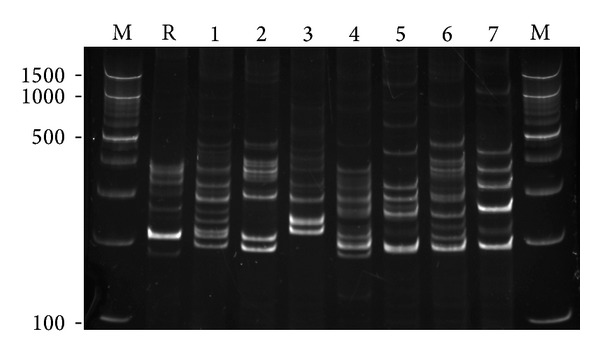
Polyacrylamide gel electrophoresis of PCR amplification products generated by FLiP analysis. Lanes 1–7: *M. tuberculosis* test strains; R: *M. tuberculosis* H37Rv; M: 100 bp DNA ladder (in base pairs).

Fast ligation-mediated PCR was applied in the present study to verify the usefulness of this method in differentiation of *M. tuberculosis* strains. The FLiP fingerprint patterns of 155 strains tested were highly variable. The number of bands in the FLiP patterns ranged from 3 to 11, with fragments between 100 and 1000 bp. The majority, 130 (84%) of the patterns, contained 5–8 bands, with an average of 6 bands.  Figure 1 shows an example of fingerprinting results for *M. tuberculosis* strains obtained with FLiP method. FLiP typing proved to be highly reproducible. The reproducibility of FLiP was assessed by independent analysis of triplicate DNA samples of 16 randomly selected test strains. Also, DNA of *M. tuberculosis* H37Rv was included as a positive control in each experiment. The FLiP patterns obtained for the reference strain and test strains in independent reactions were identical, confirming the reproducibility of this method (data not shown). However, weak (Figure 1, lane 7) and/or smear-like bands (Figure 1, lanes 2, 4, and 6) were sometimes visualized. Therefore, the unweighted pair group method with arithmetic averages (UPGMA) and Dice similarity coefficient based on manual indication of bands was used for the comparative analysis of FLiP patterns instead of a densitometry-based algorithm.

**Figure 2:**
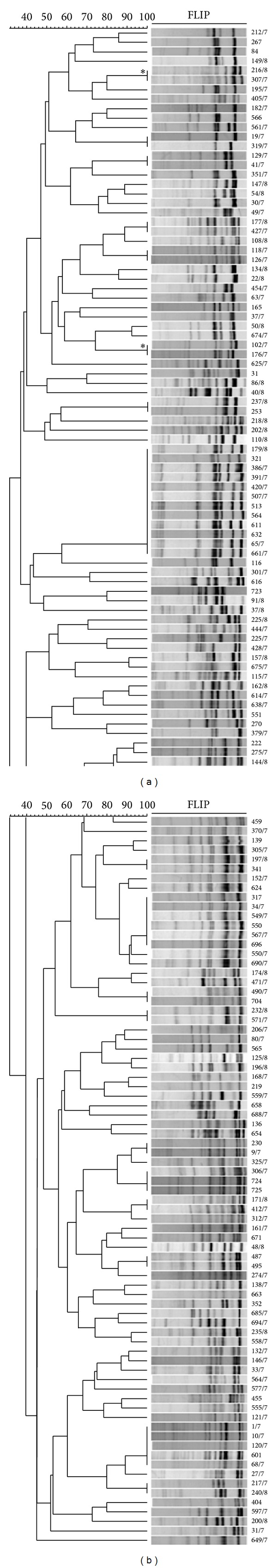
FLiP profiles of the 155 *M. tuberculosis* strains and the corresponding dendrogram. The similarity among the profiles is given as a percentage above the dendrogram. The numbers of strains are shown at the right side. Asterisks indicate two clusters of identical isolates from the same patients.

FLiP typing identified 119 distinct patterns among 155 tested strains (Figure 2). One hundred and one (65%) strains showed unique band patterns, and remaining 54 (35%) strains clustered into 18 groups containing 2–12 identical genetic profiles representing tested strains. The largest cluster was composed of 12 strains. Also, three larger clusters of strains were detected, each containing six, five, and three strains, respectively. Majority of the clusters (14 out 18) were made up of two strains each. Patterns detected in two strains isolated from the same patient at different times were identical and clustered together, further confirming stability and reproducibility of the FLiP profiles (Figure 2). The overall degree of strain differentiation generated by FLiP was 0.768 and it was higher in comparison with 0.697 observed for the same set of strains when characterized by 15-locus MIRU-VNTR typing.

Previously performed, MIRU-VNTR analysis [21] identified 108 distinct profiles among the 155 strains tested. Eighty-one (52%) strains were unique, and 74 (48%) strains were grouped in 27 clusters including 2–11 isolates. Eleven strains were grouped into the largest cluster with identical MIRU-VNTR patterns. Three large clusters of strains were also identified, each containing seven, six, and four strains, respectively. The remaining 23 clusters were composed of two strains each. As in FLiP, the repetitive isolates from two patients were identical to the first isolate also in MIRU-VNTR typing.

Clustering results from both methods were compared to determine their discriminatory power. FLiP gave resolving power only slightly higher than the 15-locus MIRU-VNTR analysis (HGDI = 0.991 and 0.990, resp.). However, detailed comparison of the clusters defined by each of the methods revealed a more apparent difference in the discriminatory abilities that favored FLiP. As shown in Table 1, 13 (48%) of the 27 MIRU-VNTR clusters included strains with multiple FLiP patterns, whereas only 4 (22%) out of the 18 FLiP clusters could be subdivided by MIRU-VNTR analysis.

The simultaneous use of both methods identified 42 isolates that clustered in 15 combined FLiP/MIRU-VNTR groups. Previously obtained IS*6110* RFLP data [21] revealed one unique pattern in each of three combined clusters consisting of seven, four, and three strains, respectively. The differences in IS*6110* RFLP patterns within the three subdivided FLiP/MIRU-VNTR clusters were, however, not significant and limited only to the presence of one or two additional IS*6110* hybridizing bands (data not shown).

## 4. Discussion and Conclusion

FLiP is a DNA typing method for *M. tuberculosis* complex strains described by Reisig et al. in 2005 [16]. It is based on the original mixed-linker approach that used one primer specific for IS*6110* and a second primer complementary to a linker ligated to the *Hha*I restriction fragments of genomic DNA in a PCR amplification. One strand of the linker molecule contained uracil instead of thymidine, and it was split by UDG assuring specificity of the reaction [17]. The mixed-linker method was successfully applied to outbreak investigations and population-based studies [17, 18, 23]. However, it requires reamplification that increases the risk of cross-contamination and multiple hands-on steps. To overcome those limitations, Reisig et al. constructed new linker oligonucleotides. That modification enabled specific amplification of RFLP fragments carrying IS*6110* after a single PCR step, following simultaneous restriction-ligation reactions, resulting in a simplified and faster typing of *M. tuberculosis* strains [16]. Kremer et al., in an extensive interlaboratory study comparing nine PCR-based assays, concluded that MIRU-VNTR and FLiP methods are both rapid, highly reliable, and discriminative epidemiological typing tools for *M. tuberculosis* [18]. However, in spite of its potential to differentiate between strains, FLiP has not been tested in molecular analyses since its publication.

To address this lack of data, we previously used FLiP to estimate molecular relationships among small sets of clinical *M. tuberculosis* isolates [19, 20]. In this study, we extend our preliminary observations and report a molecular characterization of 155 *M. tuberculosis* strains from Łódź, Poland, through genotyping by FLiP in comparison with previous results of 15-locus MIRU-VNTR analysis [21]. In accordance with our earlier reports [19, 20], FLiP patterns obtained in the present study consisted of 3–11 DNA fragments, with an average of 6 bands. This is slightly lower compared to 0–16 bands (8 average) described by Reisig et al. [16]. These authors did not comment on concordance between the numbers of IS*6110* copies detected by FLiP and IS*6110* RFLP. However, we previously noticed 1–8 bands less in 90% of FLiP patterns in comparison with IS*6110* RFLP profiles of the respective strains [20]. Also, Prod'hom et al. reported that the number of bands generated in ligation-mediated PCR, a DNA typing technique related to mixed-linker amplification, was equal to or lower than IS*6110* copy number in the strains tested [24]. These results indicate that the number of bands in the FLiP DNA fingerprints may not necessarily reflect the number of IS*6110* copies in *M. tuberculosis *strains and rather should not be identified with them. Some limitation of FLiP, inherent to banding profiles-based methods, could be occasional appearance of weak and/or smear-like bands within a pattern. However, despite this potential difficulty, FLiP still proved to be fully reproducible confirming previous observations [18, 20].

FLiP typing revealed a high degree of polymorphism among the 155 strains investigated here confirming previous data of Kremer et al. [18], although its discriminatory power was somewhat lower in the present study (HGDI = 0.991 versus 0.994). The difference was most probably due to various geographical origins of the 90 *M. tuberculosis* strains tested earlier that resulted in lower degree of clustering compared to the present study on the strains originating from a single town only. Nevertheless, FLiP was still more discriminative than 15-locus MIRU-VNTR typing (HGDI = 0.990). On the other hand, Kremer et al. observed slightly lower discriminatory potential of FLiP compared to MIRU-VNTR analysis based on 12 loci (HGDI = 0.995) [18].

Strain resolution by MIRU-VNTR typing approaches that of the reference method but varies according to the loci analyzed and between strain families [18, 25–29]. Moreover, the exclusive use of this method leads to misinterpretations of epidemiological links among *M. tuberculosis* isolates [29, 30]. Methods based on IS*6110* and methods based on MIRU-VNTR polymorphisms detect changes in different regions of the chromosome (mobile versus core); therefore clustering generated by the two types of methods does not have to be identical. In the present study, only 7% (3/42) of the strains clustered both by MIRU-VNTR and FLiP methods could be subdivided by the reference IS*6110* RFLP. These results further confirm a high discriminatory ability of FLiP and its usefulness as a secondary typing technique for verification of MIRU-VNTR clusters.

## Figures and Tables

**Table 1:** Comparison of clustering in *M. tuberculosis* strains by the use of 15-locus MIRU-VNTR and FLiP typing methods.

MIRU-VNTR		FLiP pattern (no. of strains)	MIRU-VNTR		FLiP pattern (no. of strains)
Pattern (no. of strains)	Numerical code^a^		Pattern (no. of strains)	Numerical code^a^	
1 (11)	453531333243437	1 (6), 2 (1), 3 (1), 4 (2), 5 (1), 6 (1)	16 (2) 17 (2)	423533332242325 432433343242325	30 (1), 31 (1) 32 (2)
2 (7)	482132544343228	7 (12)^b^	18 (2)	443433343242225	33 (2)
3 (6)	453533333443335	8 (3), 9 (1), 10 (1), 11 (1)	19 (2) 20 (2)	423434343242526 433334343242626	34 (1), 35 (1) 36 (2)
4 (4)	343541333431446	12 (5)	21 (2)	432335343242525	37 (2)
5 (2)	443533333443437	13 (2)	22 (2)^d^	431535342232322	38 (2)^d^
6 (2)	453533333443337	14 (2)	23 (2)	443444333443545	39 (2)
7 (2)	432433333443437	15 (1), 16 (1)	24 (2)	3112132345443138	40 (1), 41 (1)
8 (2)	463634333443637	17 (1), 18 (1)	25 (2)	4102123454443226	42 (2)
9 (2)	433533333443637	19 (1), 20 (1)	26 (2)	492134544443138	43 (1), 44 (1)
10 (2)^c^	442431542132437	21 (2)^c^	27 (2)	482532544343228	7 (2)^b^
11 (2)	453531333243237	7 (1)^b^, 22 (1)	28 (1), 29 (1)	453533333443345,	45 (2)
12 (2)	453531333443437	23 (2)		453533333443342	
13 (2)	463633333443235	24 (1), 25 (1)	30 (1), 31 (1)	453533333475347,	46 (2)
14 (2)	423432342242425	26 (1), 27 (1)		453543333463248	
15 (2)	432532342242425	28 (1), 29 (1)			

^a^Number of copies > 9 per locus is underlined.

^b^Members (*n* = 12) of the same cluster (FLiP pattern 7).

^c, d^Sequential isolates from two patients.
